# Identifying influential nodes in complex networks using a gravity model based on the H-index method

**DOI:** 10.1038/s41598-023-43585-x

**Published:** 2023-09-29

**Authors:** Siqi Zhu, Jie Zhan, Xing Li

**Affiliations:** Physical and Electronic Sciences College, Hunan University of Science and Technology of China, Xiangtan, 411100 People’s Republic of China

**Keywords:** Engineering, Mathematics and computing, Physics

## Abstract

Identifying influential spreaders in complex networks is a widely discussed topic in the field of network science. Numerous methods have been proposed to rank key nodes in the network, and while gravity-based models often perform well, most existing gravity-based methods either rely on node degree, k-shell values, or a combination of both to differentiate node importance without considering the overall impact of neighboring nodes. Relying solely on a node's individual characteristics to identify influential spreaders has proven to be insufficient. To address this issue, we propose a new gravity centrality method called HVGC, based on the H-index. Our approach considers the impact of neighboring nodes, path information between nodes, and the positional information of nodes within the network. Additionally, it is better able to identify nodes with smaller k-shell values that act as bridges between different parts of the network, making it a more reasonable measure compared to previous gravity centrality methods. We conducted several experiments on 10 real networks and observed that our method outperformed previously proposed methods in evaluating the importance of nodes in complex networks.

## Introduction

Complex networks are a pervasive presence in various domains of both human society and the natural world. In each system, individuals and their relationships can be represented as networks consisting of nodes and edges^1,2^. Recently, the identification of significant nodes in complex networks has gained significant attention from researchers, providing a new perspective for understanding the objective world and facilitating a better comprehension of the spread of diseases^3–5^, power grid protection^6^, information dissemination^7–9^, protein discovery^10^, and immunization strategies^11,12^, among other fields^13–15^.

To date, numerous centrality methods have been proposed to detect key nodes in complex networks. Centrality measurement methods can be primarily categorised into three types: local indices, global indices, and hybrid indices^16^. Local-index-based centrality methods include classical measures such as degree centrality^17^ (DC) and H-index^18^. Local-index-based methods have low computational complexity and are suitable for large-scale networks as they only consider the local neighbourhood information of nodes. However, their ability to identify influential nodes that are not central but have high impact is limited. To address this limitation, many researchers have proposed improvements, such as extended H-index centrality^19^ (EHC) and local clustering H-index centrality^20^ (LCH) methods. Global-index-based centrality methods assess individuals' influence by considering the global structural information of the network, such as closeness centrality^21^ (CC) and betweenness centrality^22^ (BC). The main drawbacks of these measurement methods are their high computational complexity and inapplicability to large-scale networks^23^. Among them, the K-shell decomposition method^24^ (KS), as a global approach, determines the influence of nodes by differentiating their core levels and operates at a faster speed. However, the main limitation of k-shell is that it assigns the same k-shell value to many nodes, resulting in low differentiation in node influence ranking. Many efforts have been made to address this issue, such as extended neighbourhood coreness^25^ (CNC+), classifying neighbourhood^26^ (CN), k-shell iteration factor^27^ (KSIF), and Mixed Degree Decomposition^28^ (MDD). The primary limitations of these global methods are their typically high computational costs as they consider the entire topological structure of the network. Hybrid-index-based centrality methods, such as local and global influence^29^ (LGI), local and global centrality^30^ (LGC) and global and local information^31^ (GLI) integrate both local and global information about nodes, aiming to strike a balance between algorithm accuracy and computational complexity.

The gravity model not only considers the attributes held by two nodes but also takes into account the shortest path information between nodes, which represents their mutual interactions and provides a basis for integrating local and global information. Inspired by this formula, Ma et al.^32^ proposed two models (G and G+) based on the gravity formula. These models adopt the k-shell value of a node as its mass and use the shortest path distance between two nodes as the distance. Building upon this, Wang et al.^33^ improved the model by considering the degree values of neighbouring nodes, resulting in the improved gravity centrality (IGC). Li et al.^34^ introduced the gravity model (GM), which employs the degree of nodes as their mass, and developed the local gravity model (LGM), which only considers node pairs within a truncated radius. Furthermore, Li et al.^35^ combined the local clustering coefficient and degree value as the mass of nodes, proposing the generalized gravity centrality (GGC). In addition, Yang et al.^36^ introduced a gravity centrality (KSGC) based on the K-shell value of nodes, considering the variations in interactions when nodes are located in different shell layers. Li et al.^37^ combined the k-shell value and k-shell iteration factor as the mass of nodes, presenting the DK-based gravity model (DKGM) to enhance the model's performance. Subsequently, they considered multiple features of nodes and proposed the multi-characteristics gravity model^38^ (MCGM). Liu et al.^39^ introduced the spreading entropy gravity Model (SEGM), incorporating the spreading information entropy of nodes into consideration.

From the above, we can observe that many of the gravity models mentioned are either based on node degree, related to the k-shell value, or a combination of both. However, It is not enough to evaluate the importance of a node solely on the basis of its single attributes; it is also necessary to consider the location of the node and the overall influence of neighbouring nodes on it. For instance, some nodes may have a relatively small k-shell index but possess significant influence since they act as bridges connecting different communities within the network. Similarly, there are nodes with lower degree or k-shell values compared to others but are closer to the most important nodes in the network, surrounded by highly influential nodes, as a result, their importance will also be enhanced. To address this issue, we propose the H-index-based gravity centrality method (HVGC), which not only considers the path information of nodes but also incorporates the overall influence of neighbouring nodes, structural hole position information of nodes, and the differential gravitational impact of nodes positioned at different locations. Experimental results demonstrate that our proposed method exhibits significant competitiveness compared to other advanced gravity models, Particularly in networks with evident community structures, it exhibits outstanding accuracy, unlike other algorithms that are prone to identifying false core nodes.

## Preliminaries

### Centrality measures

In the context of an undirected and unweighted simple network $G \, = < V, \, E >$,$V$ and $E$ respectively represent the sets of nodes and links. The cardinality of $V$ and $E$ can be expressed as $\left| V \right| = N$ and $\left| E \right| = M$, indicating the presence of $N$ nodes and $M$ links within the network. The network's connectivity structure is typically captured by its adjacency matrix $A = (a_{ij} )_{N \times N}$, where $a_{ij} = 1$ if node $i$ and node $j$ are linked, and 0 otherwise.

Degree centrality^17^ of node $i$ is defined as

1 $$ DC(i) \, = \, k(i), $$

where $$k(i) = \sum\limits_{j = 1}^{N} {a_{ij} } .$$

The maximum integer fulfilling that there are at least $H(i)$ neighbors of node $i$ whose degrees are all at least $H(i)$, represented by $H(i)$, is known as the H-index^18^ of the node $i$.

The k-shell decomposition method^24^(KS), operates through an iterative process of decomposing the network into distinct shells. Initially, KS removes nodes with a degree of 1 from the network, resulting in a decrease in the degree values of the remaining nodes. This process is repeated by removing nodes with residual degrees less than or equal to 1 until all remaining nodes have residual degrees greater than 1. The nodes removed in the first step constitute the 1-shell, and their k-shell values are assigned as 1. This process is then iteratively applied to obtain the 2-shell, 3-shell, and so on. The decomposition process continues until all nodes in the network have been accounted for.

Gravity centrality^32^ (G) of node $i$ is defined as

2 $$ G(i) = \sum\limits_{{j \in \psi_{i} }} {\frac{{k_{s} (i)k_{s} (j)}}{{d^{2} (i,j)}}} , $$

where $k_{s} (i)$ is the k-shell value of node $i$, $d(i,j)$ is the shortest path distance from node $i$ to node $j$, and $\psi_{i}$ is the set of nodes whose distance from node $i$ does not exceed 3.

Extended gravity centrality^32^ (G+) of node $i$ is described as

3 $$ G_{ + } (i) = \sum\limits_{{j \in \Lambda_{i} }} G (j), $$

$\Lambda_{i}$ is the nearest neighborhood of node $i$.

The improved gravity centrality^33^ (IGC) of node $i$ is measured by

4 $$ IGC(i) = \sum\limits_{d(i,j) \le R,j \ne i} {\frac{{k_{s} (i)k(j)}}{{d^{2} (i,j)}}} , $$

where $R$ is the truncation radius, and the optimal truncation radius $R^{*}$ can be estimated by

5 $$ R^{*} \approx \frac{1}{2}\langle d\rangle , $$

where $\langle d\rangle$ is the average distance of the network.

Extended improved gravity centrality^33^ (IGG+) of node $i$ is described as

6 $$ IGC_{ + } (i) = \sum\limits_{{j \in \Lambda_{i} }} {IGC} (j), $$

$\Lambda_{i}$ is the nearest neighborhood of node $i$.

The local gravity model^34^ (LGM) of node $i$ is determined by

7 $$ LGM(i) = \sum\limits_{d(i,j) \le R,j \ne i} {\frac{k(i)k(j)}{{d^{2} (i,j)}}} , $$

The generalized gravity centrality^35^ (GGC) of node $i$ is defined as

8 $$ GGC(i) = \sum\limits_{d(i,j) \le R,j \ne i} {\frac{{S_{p} (i)S_{p} (j)}}{{d^{2} (i,j)}}} , $$

9 $$ S_{p} (i) = {\text{e}}^{{ - \alpha C_{i} }} \times k(i) $$

10 $$ C_{i} = \frac{{2n_{i} }}{k(i)(k(i) - 1)} $$

where $C_{i}$ is the local clustering coefficient of node $i$, $n_{i}$ denotes the number of edges between neighbors of node $i$, and $\alpha = 2$.

The k-shell based on gravity centrality^36^ (KSGC) is defined as

11 $$ KSGC(i) = \sum\limits_{d(i,j) \le R,j \ne i} {F(i,j)} , $$

12 $$ F(i,j) = c_{ij} \frac{k(i)k(j)}{{d^{2} (i,j)}}, $$

13 $$ c_{ij} = e^{{\frac{ks(i) - ks(j)}{{ks_{\max } - ks_{\min } }}}} $$

where $c_{ij}$ is the coefficient of attraction exerted by node $i$ on node $j$, $k_{s} (i)$ and $k_{s} (j)$ denote the k-shell values of node $i$ and node $j$, respectively. $ks_{\max }$ and $ks_{\min }$ refer to the largest and smallest k-shell values present in the network. $d(i,j)$ is the shortest path distance from node $i$ to node $j$.

The DK-based gravity model^37^ (DKGM) is measured by

14 $$ DKGM(i) = \sum\limits_{d(i,j) \le R,j \ne i} {\frac{DK(i)DK(j)}{{d^{2} (i,j)}}} , $$

15 $$ DK(i) = k(i) + k_{s}^{*} (i), $$

16 $$ k_{s}^{*} (i) = k_{s} (i) + \frac{p(i)}{{q(k) + 1}}, $$

assume that the value of the k-shell of node $i$ is $k_{s} (i).$ For the process of the k-degree iteration, the total iteration number is $q(k)$, and node $i$ is removed in the $p(i)$ iteration of the k-degree process. $k_{s}^{*} (i)$ is called the improved k-shell index of node $i$.

The multi-characteristics gravity model^38^ (MCGM) is measured by

17 $$ {\text{MCGM}} (i) = \sum\limits_{d(i,j) \le R,j \ne i} {\frac{{\left( {\frac{k(i)}{{k_{\max } }} + \frac{{\alpha k_{s} (i)}}{{k_{s\max } }} + \frac{x(i)}{{x_{\max } }}} \right)\left( {\frac{k(j)}{{k_{\max } }} + \frac{{\alpha k_{s} (j)}}{{k_{s\max } }} + \frac{x(j)}{{x_{\max } }}} \right)}}{{d^{2} (i,j)}}} , $$

18 $$ \alpha = \frac{{\max \{ \frac{{k_{{{\text{mi}} d}} }}{{k_{\max } }},\frac{{x_{{{\text{mi}} d}} }}{{x_{\max } }}\} }}{{\frac{{k_{{s{\text{mi}} d}} }}{{k_{s\max } }}}}, $$

where $k_{mid}$, $k_{smid}$ and $x_{mid}$ denote the median of degree value, k-shell value and eigenvector centrality value, respectively. $k_{\max }$, $k_{s\max }$ and $x_{\max }$ denote the maximum values of degree value, k-shell value, and eigenvector centrality value.

The entropy-based gravity model^39^ (SEGM) is defined as

19 $$ SEGM(i) = \sum\limits_{d(i,j) \le R,j \ne i} {\frac{SE(i)SE(j)}{{d^{2} (i,j)}}} , $$

20 $$ SE(i) = e^{E(i)} k(i), $$

21 $$ E(i) = - \sum\limits_{j \in \Gamma (i)} {I(j)\ln I(j)} , $$

22 $$ I(i) = \frac{k(i)}{{\sum\limits_{j \in \Gamma (i)} {k(j)} }}, $$

where $E(i)$ is the information entropy of node $i$, $\Gamma (i)$ represents the set of neighboring nodes of node $i$,and $I(i)$ is the importance of node $i$.

### The SIR model used in this paper

To evaluate the ranking of impact generated by the algorithm and the simulation, we employed the widely used SIR model^40^. In the beginning, a single node in the network, referred to as the "source node," is in the infected state (I), while the remaining nodes are in the susceptible state (S). An infected node has the potential to infect its susceptible neighbors with a probability of $\beta$, and the probability of each infected node entering the recovery (R) state is $\lambda$, after which it ceases to participate in the dynamics. This propagation process continues until no infected nodes remain in the network. The impact of any given node $i$ can be estimated by

23 $$ F(i) = N_{r} /N $$

the number of nodes that recover after the diffusion process has stabilized is represented by $N_{r}$. For the sake of simplicity,$\lambda$ has been set to 1. Subsequently, the corresponding epidemic threshold^41^ can be computed by

24 $$ \beta_{c} \approx \frac{\langle k\rangle }{{\langle k^{2} \rangle - \langle k\rangle }} $$

where $\langle k\rangle$ and $\langle k^{2} \rangle$ are the degree distribution's average degree and second-order moments.

## Measures

### Kendall’s tau coefficient

Kendall's tau coefficient^42^ is a measure of correlation between two sequences, with a larger value indicating a greater similarity between the sequences. The definition of Kendall's tau coefficient is as follows: given two sequences $X$ and $Y$ of the same length, where the $i$ th values are represented by $x_{i}$ and $y_{i}$, respectively. Let each pair of elements $x_{i}$ and $y_{i}$ form a set, denoted by $(x_{i} ,y_{i} )$. If $x_{i} > x_{j}$ and $y_{i} > y_{j}$, or $x_{i} < x_{j}$ and $y_{i} < y_{j}$, the pairs $(x_{i} ,y_{i} )$ and $(x_{j} ,y_{j} )$ are considered concordant. They are considered discordant if $x_{i} > x_{j}$ and $y_{i} < y_{j}$, or $x_{i} < x_{j}$ and $y_{i} > y_{j}$. If $x_{i} = x_{j}$ and $y_{i} = y_{j}$, the pair is neither concordant nor discordant. Therefore, the Kendall's tau coefficient τ is defined as

25 $$ \tau = \frac{{2(n_{ + } - n_{ - } )}}{N(N - 1)} $$

where $n_{ + }$ is the number of concordant pairs, and $n_{ - }$ is the number of discordant pairs.

### Jaccard similarity coefficient

In some applications, concentrating on the top-rank nodes rather than all nodes may be appropriate. In contrast to the Kendall correlation coefficient, the Jaccard similarity coefficient is utilized to assess the similarity between the top-k nodes in two ranking lists^25,43^. The Jaccard similarity is calculated by dividing the number of common nodes by the number of unique nodes in the two lists, and its expression is

26 $$ Jaccard(X,Y) = \frac{|X \cap Y|}{{|X \cup Y|}} $$

where $X$ and $Y$ represent the top-k nodes with the highest influence as determined by two different methods. In the context of our experiments, $X$ represents the top-k nodes identified by HvGC and other baseline methods, while $Y$ represents the top-k nodes obtained through the SIR simulation. We use the Jaccard similarity coefficient to measure the similarity between these two sets of top-k nodes. The Jaccard similarity coefficient ranges from 0 to 1, where a higher value indicates a greater degree of similarity between the two ranking results. A Jaccard similarity coefficient of 0 indicates completely distinct results, while a value of 1 indicates that the two sets of top-k nodes are identical.

### The monotonicity index

The monotonicity^25^ $M$ is used to quantitatively measure the resolution of different indices in ranking list $X$, and can be calculated by

27 $$ M(X) = \left[ {1 - \frac{{\sum\limits_{c \in V} {N_{c} } \left( {N_{c} - 1} \right)}}{N(N - 1)}} \right]^{2} $$

where $N$ is the size of network, and $N_{c}$ is the number of nodes with the same index value $c$.

## Results

### Algorithms

**Figure 1:**
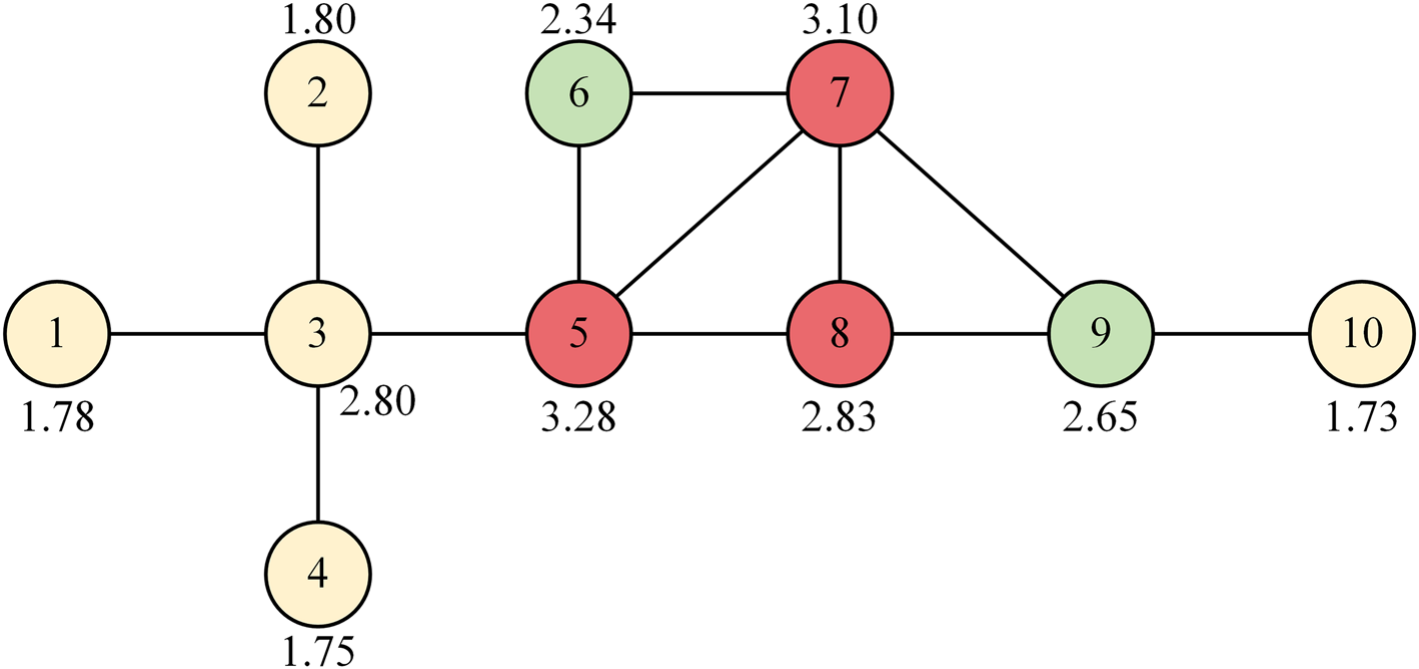
A toy network. The red node is ranked first in terms of H-index, while green and yellow represent second and third, respectively.

Previous research has utilized the gravity model approach to analyze node importance in complex networks. Degree and k-shell values are commonly used metrics to consider the number of neighbors a node has and its position within the network, respectively. However, these metrics alone do not capture the overall influence of a node's neighbors. While the H-index considers the importance of a node's neighbors, it may overlook certain information from neighboring nodes, failing to account for the collective impact of all neighbors. We take the toy network shown in Fig. 1 to illustrate the problem for H-index, where the node spreading capacity derived from 1000 independent runs of the SIR model has been numerically labeled in Fig. 1. Obviously, $H(1) = H(2) = H(3) = H(4) = H(10) = 1$, $H(5) = H(7) = H(8) = 3$,$H(6) = H(9) = 2$, where $H(i)$ represents the H-index of node $i$. The H-index always assigns the same value to different nodes, which leads to a lack of excellence in the ability to differentiate the influence of nodes.

The same issue exists in DC^17^ and KS^24^. Additionally, from Fig. 1, it can be observed that Node 3 has a higher propagation capability compared to Node 9, but Node 3 has a lower H-index than Node 9. This indicates that the H-index overlooks some information from the neighbors of a node. From this, we take out all neighboring nodes in the set of neighbors of node $i$ with degree values greater than or equal to $H(i)$ and add up the degree values of these nodes to measure the overall influence of the neighboring nodes on node $i$. The value obtained is denoted as $HV(i)$, and the expression is

28 $$ HV(i) = \sum\limits_{{j \in \Lambda_{i} }} {\left[ {k(j)|k(j) \ge H(i)} \right]} , $$

where $\Lambda_{i}$ is the nearest neighborhood of node $i$,$H(i)$ represents the H-index of node $i$.

By incorporating the overall influence of node neighbors into the definition, it enhances the discriminative power of node identification compared to the H-index. However, it is still insufficient to accurately distinguish cluster-like nodes, due to their close connections, these nodes can more easily achieve greater HV values, but, their actual influence may not be greater than that of nodes with lower HV values, As shown in Fig. 1. $HV(6) = 8$,$HV(9) = 7$,$HV(3) = 4$, and the actual propagation capacity from high to low is nodes 3, 9, and 6, a similar problem with the k-shell approach was noted by Liu et al.^44^ In other words, removing node 3 from the network would result in nodes 1, 2, and 4 losing their interactions with the core nodes, while removing node 6 has a minimal impact on information transmission in the network. This finding demonstrates the higher importance of nodes that serve as bridges between different clusters compared to those within individual clusters.

Based on this, we considered the structural hole position of nodes to enhance the algorithm's ability to identify nodes within community networks. This allows us to identify those bridge nodes that may not have high HV values but play a crucial role in facilitating information flow across different parts of the network. The network constraint coefficient measures the level of constraints imposed on nodes forming a structural hole (SH) in a network^45^, and it can be calculated as follows:

29 $$ c(i) = \sum\limits_{j \in \Gamma (i)} {\left( {p_{ij} + \sum\limits_{w \in \Gamma (j) \cap \Gamma (i)} {p_{iw} } \cdot p_{wj} } \right)^{2} } , $$

30 $$ p_{ij} = \frac{{z_{ij} }}{{\sum\limits_{w \in \Gamma (i)} {z_{iw} } }}, $$

where $\Gamma (i)$ represents the set of neighboring nodes of node $i$, and $w \in \Gamma (i) \cap \Gamma (j)$ indicates the nodes that are common neighbors of both node $i$ and node $j$. $p_{ij}$ represents the proportion of energy invested by node $i$ to maintain its relationship with node $j$. where $z_{ij} = 1 \, (i \ne j)$ if there is a link between nodes $i$ and $j$, otherwise $z_{ij} = 0$. Based on the above discussions, the gravity centrality based on the H-index (HVGC) measure proposed in this paper is defined as follows:

31 $$ HVGC(i) = \sum\limits_{d(i,j) \le R,j \ne i} {e^{ - c(i)} \frac{Hv(i)Hv(j)}{{d^{2} (i,j)}}} , $$

where $c(i)$ represents the structural hole constraint coefficient in Eq. (29). A smaller value of $c(i)$ indicates that the node occupies more structural holes and has a stronger ability to bridge different parts of the network. Finally, the metrics, including HVGC, H-index, HV, DC, and KS, were computed for each node in the toy network and compared with the node's spreading capability (SC). The results are presented in Table 1, revealing that HVGC achieves a nearly identical ranking to SC, indicating excellent performance. The algorithmic description of the HVGC is provided in Algorithm 1.

**Table 1:** The ranking results of SIR, DC, KS, H-index, HV, and HVGC on the toy network.

Node	SC	DC	KS	H-index	HV	HVGC
5	3.28	4	2	3	11	248.02
7	3.10	4	2	3	10	208.09
8	2.83	3	2	3	11	164.73
3	2.80	4	1	1	7	125.38
9	2.65	3	2	2	7	103.72
6	2.34	2	2	2	8	76.91
2	1.80	1	1	1	4	10.30
1	1.78	1	1	1	4	10.30
4	1.75	1	1	1	4	10.30
10	1.73	1	1	1	3	7.72

**Figure 2:**
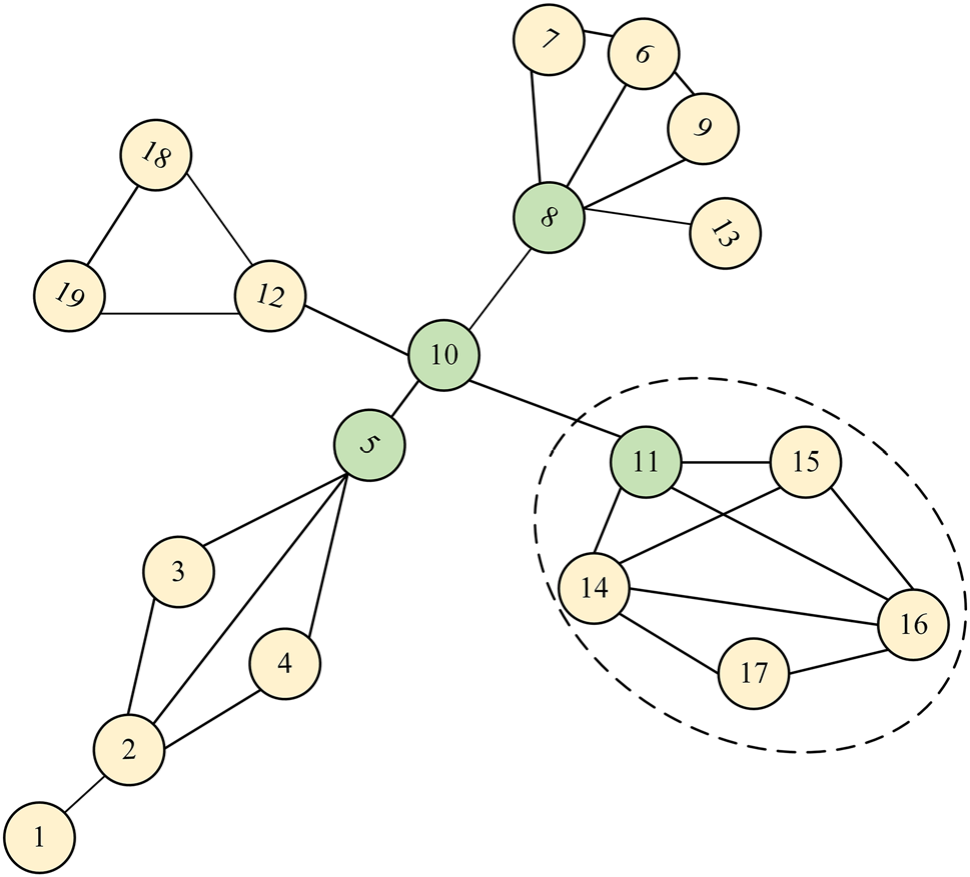
A sample network with an obvious community structure, where the four nodes with the strongest propagation capabilities are marked in green.

In addition, Fig. 2 depicts a network with a clear community structure, where the four nodes with the strongest propagation capabilities are marked in green. The propagation capabilities of these nodes were determined through 1000 independent experiments using the SIR model. We compared HVGC with other gravity model-based methods in identifying the top 5 nodes in this network, and the results are presented in Table 2. 
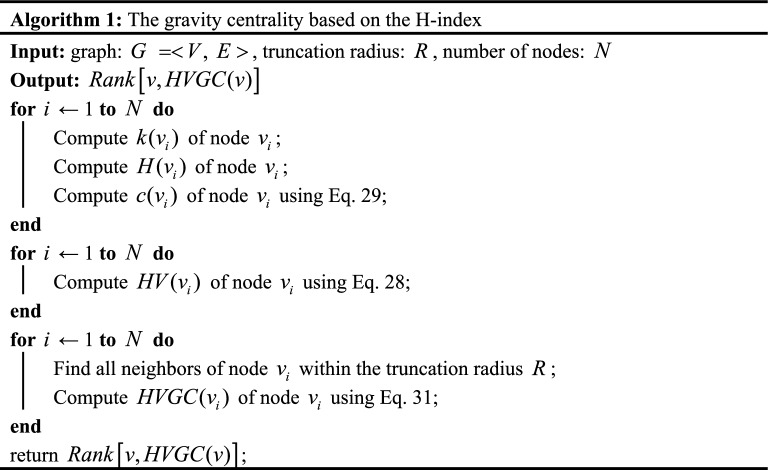

**Table 2:** Comparison of the rankings of the top-5 nodes identified by different methods and the rankings based on the SIR propagation ability in the sample network.

Rank	SIR	G+	IGC+	LGM	GGC	KSGC	DKGM	MCGM	SEGM	HVGC
1	10	11	11	10	10	11	11	11	10	10
2	11	14	14	8	8	8	14	16	11	11
3	8	16	16	11	5	10	16	14	14	8
4	5	15	10	14	11	14	10	10	16	5
5	16	10	15	16	2	16	8	15	8	16

### Data description

This paper evaluates the efficacy of HVGC by analyzing ten real networks from six distinct domains, including a transportation network(USAir^46^), an infrastructure network (Power^47^), a communication network (Email^48^), a technology network (Router^49^), two collaborative networks (Jazz^50^and NS^51^), and four social networks (Facebook^52^, PB^53^, WV^54^, and Sex^55^). Table 3 presents the fundamental topological properties of these networks. $N$ represents the number of nodes in the network, and $M$ represents the number of links. The average degree of nodes is denoted as $\langle k\rangle$, and the average distance between pairs of nodes is denoted as $\langle d\rangle$. The clustering coefficient^47^ of the network is denoted by $C$, while $r$ represents the assortative coefficient^56^. The degree heterogeneity^57^ of the network is denoted by $H$. Additionally, $\beta_{c}$ represents the epidemic threshold^58^ of the SIR model^40^ used to simulate the diffusion process.

**Table 3:** The topological features of ten real networks.

Networks	$N$	$M$	$\langle k\rangle$	$\langle d\rangle$	$C$	$r$	$H$	$\beta_{c}$
Jazz	198	2742	27.6970	2.2350	0.6334	0.0202	1.3951	0.0266
NS	379	914	4.8232	6.0419	0.7981	0.0817	1.6630	0.1424
Email	1133	5451	9.6222	3.6060	0.2540	0.0782	1.9421	0.0565
PB	1222	16,714	27.3552	2.7375	0.3600	0.2213	2.9707	0.0125
Facebook	4039	88,234	43.6910	3.6925	0.6170	0.0636	2.4392	0.0095
WV	7066	100,736	28.5129	3.2475	0.2090	0.0833	5.0992	0.0069
Sex	15,810	38,540	4.8754	5.7846	0.0000	0.1145	5.8276	0.0365
USAir	332	2126	12.8072	2.7381	0.7494	0.2079	3.4639	0.0231
Power	4941	6594	2.6691	18.9892	0.1065	0.0035	1.4504	0.3483
Router	5022	6258	2.4922	6.4488	0.0329	0.1384	5.5031	0.0786

### Empirical results

**Figure 3:**
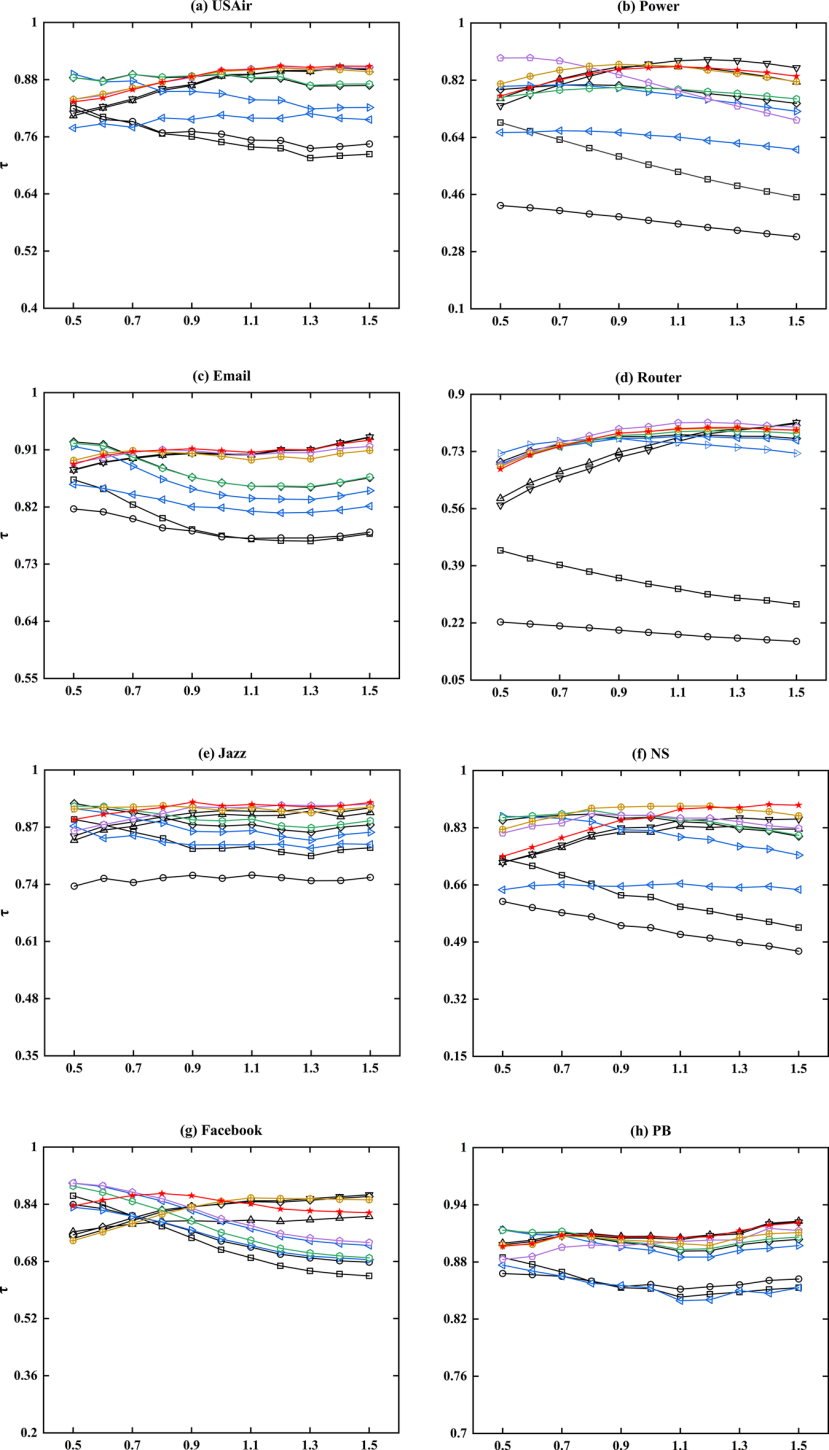

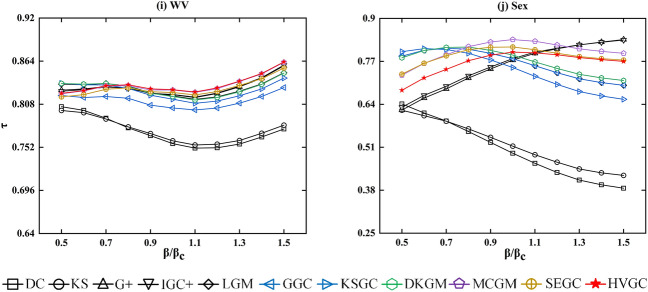
Kendall's Tau was utilized to measure the accuracy of the algorithms at various $\beta$ values. The different colour symbols represent different methods, and the red symbol represents HVGC algorithms.

Based on the aforementioned real network, we conducted simulations and compared the influence rankings of various algorithms utilizing the SIR model. In order to ensure the credibility of our findings and the standard ranking of nodes' influence, we conducted 1000 independent experiments for each given network and transmission probability $\beta$, with any one node being chosen as the seed node once during each run. The processor and runtime environment used for the calculations are i7-12700H and Python 3. The development platform used for this paper is Anaconda 3, and the code was executed in Jupyter Notebook. Kendall's tau ($\tau$) was utilized to evaluate the accuracy of the algorithms, with a higher value indicating a greater correlation between the observed sequences and an improved algorithm performance. Table 4 provides a comparison of the accuracy of the proposed algorithm (HVGC) and ten benchmark algorithms, which include degree centrality^17^ (DC), k-shell decomposition method^24^ (KS), the extended version of gravity centrality^32^ (G+), extended version of improved gravity centrality^33^ (IGC+), local gravity model^34^ (LGM), generalized gravity centrality^35^ (GGC), the improved gravitational centrality based on k-shell values^36^ (KSGC), the DK-based gravity model^37^ (DKGM), multi-characteristics gravity model^38^ (MCGM), and entropy-based gravity model^39^ (SEGM).Additionally, Fig. 3 displays the accuracy of the different algorithms for varying values of $\beta$, within the range of $0.5\beta_{c}$ to $1.5\beta_{c}$.

**Table 4:** The algorithms’ accuracies for $\beta = \beta_{c}$, measured by the Kendall’s Tau (τ).

Networks	DC	KS	G+	IGC+	LGM	GGC	KSGC	DKGM	MCGM	SEGM	HVGC
Jazz	0.8218	0.7536	0.8994	0.9077	0.8725	0.8301	0.8592	0.8843	**0.9142**	0.9084	*0.9184*
NS	0.6235	0.5327	0.8168	0.8316	0.8604	0.6604	0.8217	0.8653	**0.8671**	0.8938	0.8600
Email	0.7747	0.7730	0.9026	0.9038	0.8581	0.8189	0.8387	0.8579	**0.9046**	0.8997	*0.9088*
PB	0.8520	0.8563	**0.9060**	0.9050	0.8975	0.8529	0.8924	0.8990	0.8993	0.9016	*0.9063*
Facebook	0.7127	0.7369	0.8410	0.8400	0.7926	0.7428	0.7608	0.7994	**0.8479**	0.8387	*0.8494*
WV	0.7568	0.7604	0.8203	0.8206	0.8189	0.8031	0.8145	0.8180	**0.8263**	0.8232	*0.8268*
Sex	0.4919	0.5133	0.7738	0.7783	0.7782	0.7782	0.7514	0.7890	*0.8359*	**0.8136**	0.7980
USAir	0.7488	0.7657	0.8874	0.8901	0.8914	0.8055	**0.8505**	0.8918	0.8984	0.8971	*0.9001*
Power	0.5542	0.3786	0.8652	*0.8705*	0.7953	0.6467	0.7831	0.7938	0.8126	**0.8665**	0.8590
Router	0.3357	0.1919	0.7479	0.7342	0.7739	0.7694	0.7587	0.7808	*0.8035*	0.7894	**0.7895**

The top-ranked value in each row of the table is marked in italics, the second in bold.

**Figure 4:**
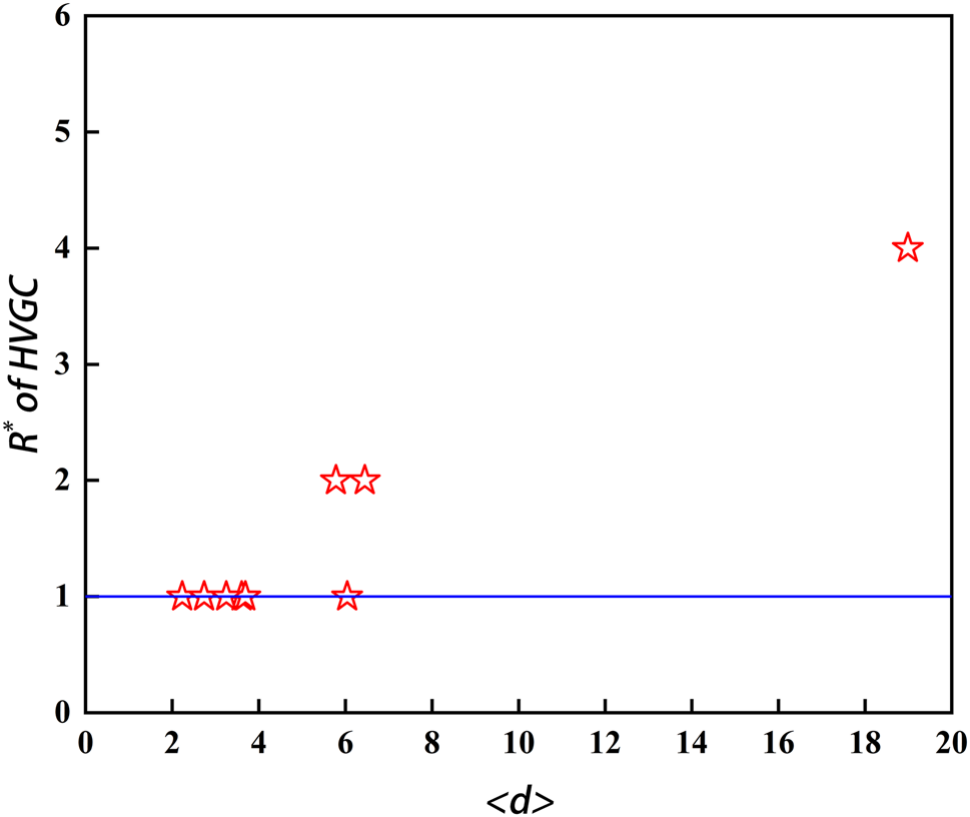
The optimal truncation radius $R^{*}$ of HVGC at $\beta = \beta_{c}$ is presented in the graph. Each pentagram in the graph corresponds to a network, with a total of ten networks represented. The blue line corresponds to $R^{*} = 1$. Specifically, for HVGC, the value of $R^{*}$ is 1 in Email, Facebook, Jazz, PB, USAir, NS and WV networks, 2 in Router and Sex networks, and 4 in Power network. The majority of the networks have an optimal truncation radius of 1, with the next most common radius being 2. This outcome aligns with the characteristics of domain centrality, which typically considers first-order and second-order neighbor nodes. HVGC represents a significant advancement over the H-index in domain centrality to obtain centrality, which is consistent with this characteristic. However, this does not impede its competitiveness relative to other algorithms, as it manages to achieve both simplicity and accuracy.

According to Table 4, the methods that utilise the gravitational formula (G+, IGC+, LGM, GGC, KSGC, DKGM, MCGM, SEGM, and HVGC) exhibit significant advantages over classical methods (DC and KS). These advantages are especially prominent in the Power, Router, NS, and Sex networks. Furthermore, it is noteworthy that among all gravity-based algorithms tested on the ten networks, HVGC exhibited the best overall performance. Its Kendall coefficient ranked first in six out of ten networks, with a remarkable 70% proportion being in the top two ranks. Specifically, HVGC ranked first in the Jazz, email, Facebook, PB, WV, and USAir networks and second in the Router network. Additionally, as shown in Fig. 3, when $\beta = \beta_{c}$, although HVGC did not perform best in the NS, Power, and Sex networks, as $\beta$ increases, its performance becomes very close to or even surpasses the previous best-performing algorithm. Taking into account HVGC's superior performance in community-type networks discussed earlier, it demonstrates a stronger overall performance, affirming the robustness of our findings. Furthermore, Fig. 4 displays the optimal truncation radius of HVGC in the ten real networks, revealing that the majority of networks concentrate their optimal truncation radius at $R = 1$. This indicates that HVGC achieves remarkably high accuracy by considering only the influence of the first-order neighbouring nodes of a node, while most other gravity model methods require considering information from second- or third-order neighbouring nodes. In other words, HVGC achieves a high level of accuracy while incurring lower time costs.

## Discussion

This paper introduces a novel method called HVGC for identifying influential nodes in a network. While the original gravity model considered both neighbourhood and path information, this new method enhances the existing gravity centrality approaches by taking into account the overall influence of a node's neighbourhood, considering the structural hole position of nodes, and incorporating the differences in interactions between nodes. This method addresses the limitations of existing gravity centrality methods and strengthens the ability to identify important nodes in networks with clear community structures. Therefore, this approach demonstrates a high level of comprehensive performance. We conducted an analysis of the SIR dynamic propagation process in 10 real networks to compare the performance of HVGC with previous state-of-the-art methods. The results, as shown in Table 4, indicate the strong competitiveness of our method.

**Figure 5:**
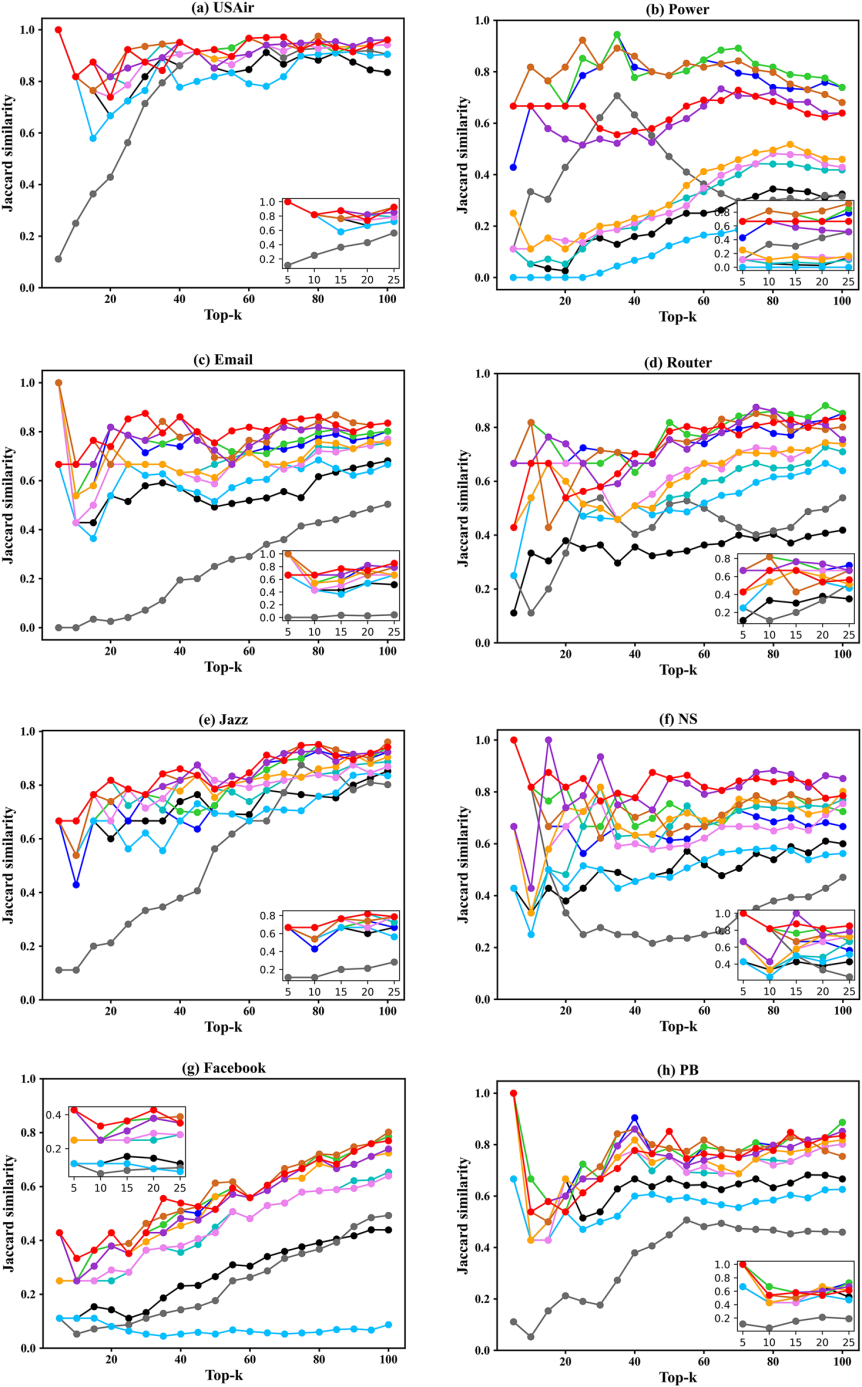

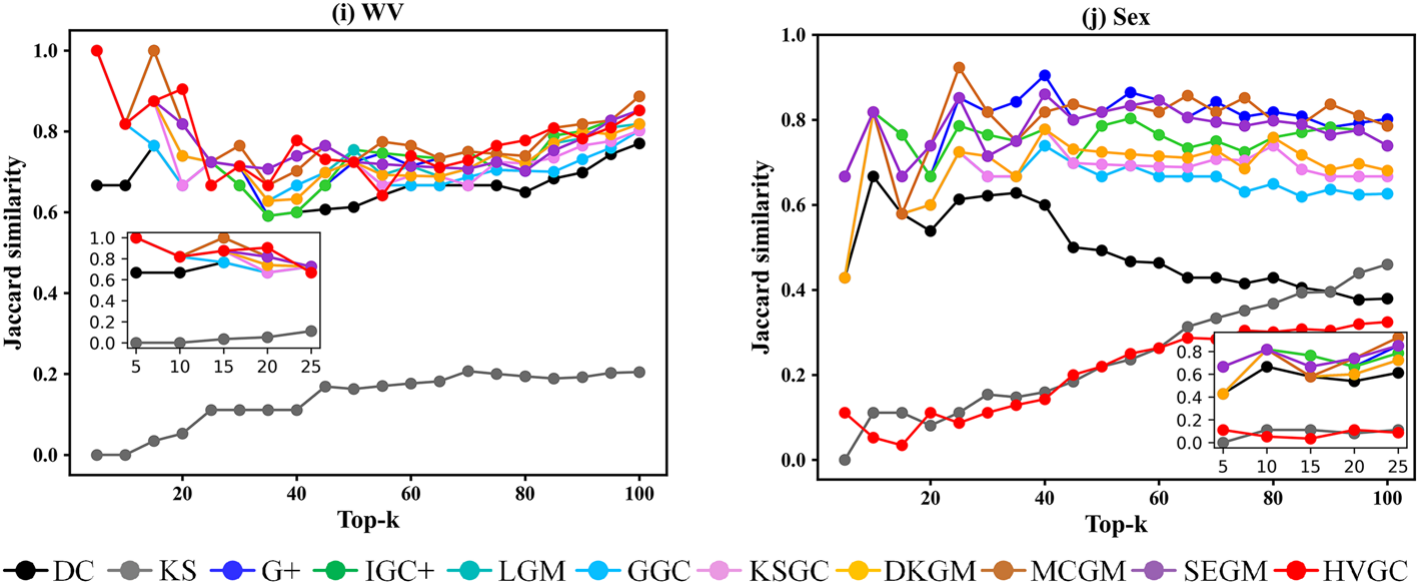
The Jaccard similarity coefficients on the top-k influential spreaders.

In certain scenarios, it is necessary to identify the top-k influential nodes for controlling information propagation. Therefore, in addition to evaluating the different ranking methods for individual nodes, we also assessed their performance in identifying the top-k influential spreaders. In other words, we compared the ranked lists of node influence obtained from the ranking methods with the ranked lists of node influence obtained from the SIR simulation, both sorted in descending order. Subsequently, we analysed the similarity between the two lists by considering the top-k nodes. Figure 5 illustrates the results of the Jaccard coefficient for identifying the top-k influential spreaders, ranging from 5 to 100 with a step size of 5. The X -axis shows the number of top influential spreaders, and the Y -axis shows the Jaccard similarity coefficients.

We can observe that, except for the Sex, Power, PB, and Router networks, HVGC exhibits the best and most stable overall performance in identifying the top-k influential spreaders in other networks. Specifically, across all networks, as the number of selected top-k nodes increases, HVGC consistently maintains a high-level or steadily increasing Jaccard coefficient, while other methods display varying degrees of fluctuations. Furthermore, we provide detailed plots for the top-25 nodes, revealing that HVGC consistently ranks among the top three in identifying the top-25 influential spreaders and, in some cases, even secures the first position, except for the Sex network. Therefore, we can conclude that HVGC not only accurately ranks the influence of all nodes in the network but also successfully identifies the top-k nodes with the highest impact.

After applying monotonicity^25^, we assessed the resolution of various algorithms. Table 5 illustrates that HVGC and MCGM demonstrate similar performance in terms of monotonicity. However, HVGC excels in the majority of networks by solely considering the first-order neighbour information of nodes, whereas MCGM, even with the inclusion of second-order neighbour information, does not necessarily outperform HVGC and incurs higher computational complexity. Furthermore, HVGC demonstrates significantly better performance in identifying important nodes in networks with community structure compared to MCGM. Therefore, overall, HVGC surpasses other gravity model algorithms. Based on the results presented in Table 5, HVGC consistently ranks either at the top or very close to the best-performing algorithm in terms of monotonicity.

**Table 5:** Monotonicity of the various algorithms is observed, with the best algorithm for each network highlighted in bold.

Networks	DC	KS	G+	IGC+	LGM	GGC	KSGC	DKGM	MCGM	SEGM	HVGC
Jazz	0.9659	0.7944	0.9993	0.9994	0.9990	0.9992	0.9992	0.9993	0.9994	0.9994	**0.9994**
NS	0.7642	0.642	0.9951	0.9951	0.9950	0.9953	0.995	0.9951	**0.9955**	0.9954	0.9955
Email	0.8873	0.8088	0.9999	0.9999	0.9976	0.9997	0.9995	0.9995	0.9999	0.9999	**0.9999**
PB	0.9328	0.9063	0.9992	0.9992	0.9991	0.9992	0.9992	0.9992	0.9993	0.9993	**0.9993**
Facebook	0.9739	0.9419	0.9998	0.9998	0.9998	0.9998	0.9998	0.9998	**0.9999**	0.9998	0.9999
WV	0.776	0.7673	0.9996	0.9996	0.9985	0.9996	0.9995	0.9993	0.9996	0.9996	**0.9996**
Sex	0.6001	0.5287	0.9996	0.9996	0.9989	0.9989	0.9996	0.9997	**0.9997**	0.9997	0.9997
USAir	0.8585	0.8114	0.9951	0.9951	0.9933	0.995	0.9942	0.9941	0.9951	0.9951	**0.9951**
Power	0.5926	0.246	0.9996	0.9997	0.9998	0.9998	0.9998	0.9998	**0.9999**	0.9998	0.9999
Router	0.2886	0.0691	0.9964	0.9964	0.9964	0.9965	0.9965	0.9965	**0.9965**	0.9966	0.9965

Based on the above discussion, it is evident that centrality based on the gravitational model is more accurate than classical centrality. However, many of these models tend to identify false core nodes in the network and do not take into account the influence of neighbouring nodes. In our proposed HVGC (H-index-based Gravity Centrality), we address this limitation by comprehensively considering the overall impact of a node's neighbours and its position within the network's structural holes. This approach effectively overcomes the drawbacks of gravity-based methods and demonstrates superior performance compared to other algorithms.

Despite the excellent performance exhibited by HVGC, it shares a common limitation with other gravity-based methods, namely the need to determine the optimal truncation radius $R$. However, this disadvantage is mitigated by the fact that most real networks exhibit small-world characteristics^47,59^, and the optimal truncation radius is approximately linearly related to the average distance^34^. Furthermore, since HVGC is derived from the domain centrality method, even considering only the first-order neighbor nodes in the ten real networks studied can lead to very high performance and accurate results.

In conclusion, while HVGC demonstrates better overall performance compared to other gravity-based methods and introduces improvements to existing gravity models, there are still areas that require further refinement. For example, the current approach does not consider the influence of weight factors associated with different indicators. Instead, it directly operates on the indicator values of the nodes. The weights of HV and the structural hole constraint coefficient $c(i)$ in the computation process may affect the accuracy of the algorithm. In networks with clear community structures, a higher weight for $c(i)$ may lead to better performance, while in other types of networks, a lower weight may yield better results. Therefore, future work may involve incorporating adjustable parameters to balance the weights of different indicators, which is a direction for further exploration. Additionally, these algorithms have not been evaluated in weighted networks, where the impact of the path from node $i$ to node $j$ may differ from that of the path from node $j$ to node $i$, and the link heterogeneity^60^ in a weighted network may result in varying node impact. Lastly, future research may involve incorporating adjustable parameters to modify the interplay of gravitational forces among nodes and balance the weights of different metrics in order to improve the performance of the algorithm.

## Data Availability

All relevant data are available at https://github.com/MLIF/Network-Data.
